# Complete Recovery of Thoracic Myelopathy after Eleven Months of Paraplegia: A Case Report

**DOI:** 10.5704/MOJ.1503.004

**Published:** 2015-03

**Authors:** S Manmohan, ZA Nor Azlin, M Fazir, A Dzulkarnain, JH Goh

**Affiliations:** Department of Orthopaedics and Traumatology, Kuala Lumpur Hospital, Kuala Lumpur, Malaysia

**Keywords:** Spine tuberculosis, late spine decompression, neurological recovery

## Abstract

Instances of neurological recovery after early decompression of the spine in non-traumatic spinal cord compression are well documented. We present a patient with paraplegia of 11 months’ duration due to atypical spinal tuberculosis who showed complete neurological recovery in three months.

## Introduction

Any tubercular spinal lesion that does not have the typical clinical or radiological features is referred to as atypical spinal tuberculosis. The importance of these lesions is that they are rare and difficult to diagnose clinically and radiographically. This may lead to delay in treatment and in certain patients, increased chances of neurologic affection. Early surgical intervention gives good neurological recovery.

## Case Report

**Fig. 1:**
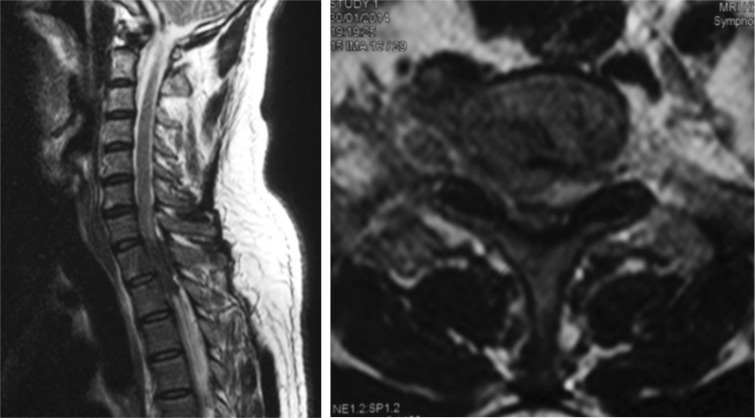
T2 weighted sagittal and axial images showing a extradural hypointense lesion which is encasing the cord and causing stenosis at the level of Thoracic 1 and 2.

**Fig. 2:**
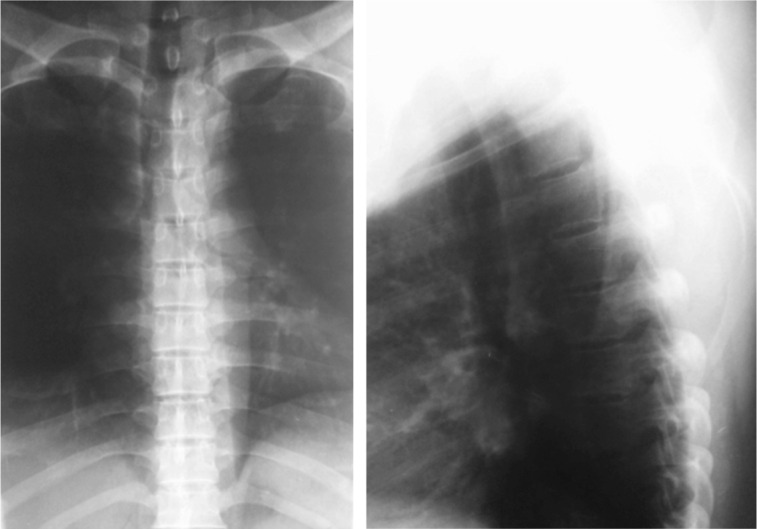
Radiographs of the thoracic spine are normal.

A 38-year old man who presented with progressive worsening of bilateral lower limbs weakness and paresthesia for one week. The weakness gradually worsened until the patient developed bowel and bladder symptoms at five months and was paraplegic at six months from time of presentation. There was no history of trauma or constitutional symptoms of malignancy and infection. MRI of the spine at five months from onset of symptoms was reported as showing a lesion at the Thoracic 1 and 2 vertebral levels (T1 and T2)(Fig 1a and Fig 1b). The plain radiographs did not show any abnormalities (Figure 2a and Figure 2b).

The initial working diagnosis was tuberculosis (TB) of the spine at T1 and T2 with neurological deficit and he was started on anti-TB chemotherapy. He was first seen at the Kuala Lumpur Hospital spine clinic at one year from the time of presentation (6 months from the time of bilateral lower limb having zero power).

On examination, the patient was a medium built man with normal neurological findings over both upper limbs. Lower limb neurological examination revealed hypertonicity at .L2 to S1 myotome with MRC grade 0 motor power. Sensory level was at T2 with pin prick testing. There was 2+ hyperreflexia, and Barbinski reflex was positive.

**Fig. 3:**
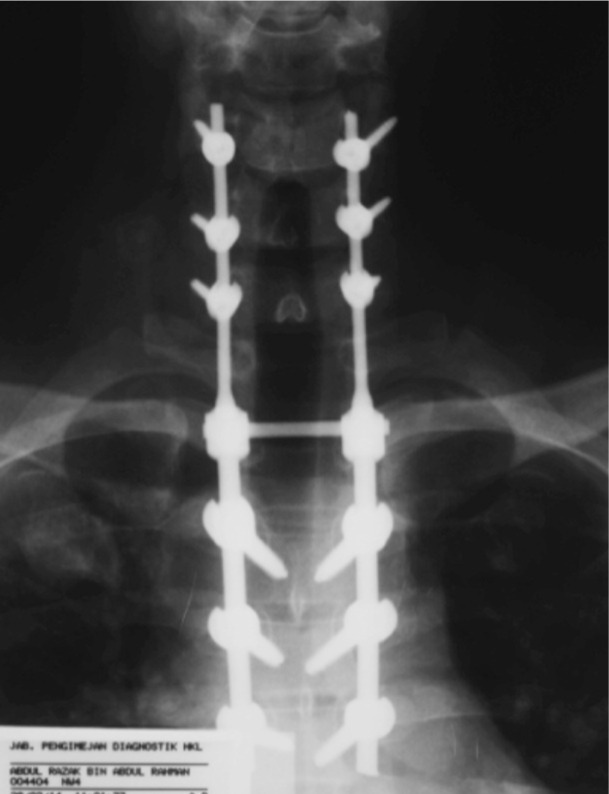
Post decompressive laminectomy T1-T2 with instrumented fusion from C5 to T5.

Haemoglobin was 17.5, White blood cell count was 8.01, ESR was 38, CRP was 5.6 and Mantoux test was negative. Since the condition was deteriorating with the chemotherapy, surgical intervention was decided as the next option He underwent decompressive laminectomy at T1-T2 with posterior spinal instrumentation and fusion from 5th cervical to 5th thoracic segments. (Figure 3)

Intra-operative, there was a flat fibrous tissue encasing the dura dorsally at the epidural space at the T1 and T2 levels. This tissue was not friable. and yielded to teasing off from the dura. There was no caseous material or pus detected. No focus of infection was found at the lamina, transverse process or facets. Histopathological examination of the tissue was reported as thickened fibrous tissue with infiltrates of lymphocytes, plasma cells and histiocytes. The tissue for Ziehl-Neelson staining was negative and PCR for TB was negative. The overall impression was that this was chronic inflammation of the dura.

By the 25th post-operative day his lower limbs power had improved to MRC grade 2/5. At review in the clinic three months after surgery he had full neurological recovery. He was able control his bladder and bowel. He returned to work as a driver six months after the spinal surgery. His anti TB chemotherapy was discontinued at 12 months after surgery.

## Conclusion

Any tubercular spinal lesion that does not have the typical clinical or radiological features is referred to as atypical spinal tuberculosis. The importance of these lesions is that they are rare and difficult to diagnose clinically and radiographically. This may lead to delay in treatment and in certain patients, increased chances of neurologic affection. The reported incidence of atypical spinal tuberculosis is about 2.1%^1^

A new classification of atypical spinal tuberculosis describes two major categories; namely, atypical radiographic presentation and atypical clinical presentation. This patient belonged to the second group where the clinical picture was one of “spinal tumor. syndrome”. The patients with spinal tuberculosis who presented with the features of both compressive myelopathy and radiculopathy, without any radiologically evident bony lesion are described as spinal tumor syndrome. The differential diagnoses are neurofibroma, meningioma, lipoma, astrocytoma and other neural tissue tumors^2^.

Patients with extradural tuberculoma present with neurologic manifestations without any spinal deformity or any evidence of osseus lesion on - plain radiographs. These lesions were referred to previously as extraosseus, extradural granulomas. Surgery was essential for decompression^3^.

To our knowledge this is the only case ever documented that showed complete neurological recovery in three months after being paraplegic for eleven months. It is not uncommon for patients to present very late in these parts of the world. Our patient presented eleven months after complete neurological deficit to our centre. MRI of the thoracic spine revealed an extradural intraspinal mass posteriorly at T1 and T2 spine. Typical MRI findings in TB spine include well defined paraspinal abnormal signal, a thin and smooth abscess wall, presence of paraspinal and intraosseous abscess, subligamentous spread to three or more vertebral levels, involvement of multiple vertebral bodies, thoracic spine involvement and hyperintense signal on T2 weighted images^4^ The MRI of this patient showed none of the above features of TB spine except for the involvement of the thoracic spine.

We could not arrive at a definitive diagnosis as all investigations were inconclusive. A provisional diagnosis of extra-osseous extradural tuberculosis of thoracic spine with myelopathy was made He was on anti TB chemotherapy for eleven months. but continued to show neurological deterioration. The option of decompression surgery was discussedwith the patient. However due to lack of literature we could not prognosticate his recovery. The decision for surgery was made at eleven months when there was complete neurological deficit. He showed complete recovery at 6 months post-operatively. We continued anti TB chemotherapy for 12 months after surgery, inspite of smear and culture being negative for Acid Fast Bacilli (AFB) in this patient. Studies have shown that smear negativity and culture negativity for AFB in spine tuberculosis was around 65% and 43% respectively^5^. We anticipated smear and culture negative for AFB in this patient as he had been on anti TB for eleven months prior to surgery.

We postulate the neurological recovery is due to the nature of the compression of the cord. The structure that was causing the compression was an encasing fibrous tissue. This tissue caused a ‘soft compression’ as opposed to an osseous compression which is a hard compression. This type of compression is seen in ossification of yellow ligament and ossification of posterior longitudinal ligament. The MRI did not show any evidence of myelomalacia, thus giving a good prognosis and recovery.

Atypical spinal tuberculosis is rare and a high index of suspicion is needed to make a diagnosis. We conclude that atypical spinal tuberculosis may benefit from surgical decompression even with late neurological presentation.
